# Evaluation of Changes in Depression, Anxiety, and Social Anxiety Using Smartphone Sensor Features: Longitudinal Cohort Study

**DOI:** 10.2196/22844

**Published:** 2021-09-03

**Authors:** Jonah Meyerhoff, Tony Liu, Konrad P Kording, Lyle H Ungar, Susan M Kaiser, Chris J Karr, David C Mohr

**Affiliations:** 1 Center for Behavioral Intervention Technologies Department of Preventive Medicine Northwestern University Chicago, IL United States; 2 Department of Computer and Information Science University of Pennsylvania Philadelphia, PA United States; 3 Department of Bioengineering University of Pennsylvania Philadelphia, PA United States; 4 Department of Neuroscience University of Pennsylvania Philadelphia, PA United States; 5 Audacious Software Chicago, IL United States

**Keywords:** mHealth, personal sensing, digital phenotyping, passive sensing, ecological momentary assessment, depression, anxiety, digital biomarkers, digital phenotyping, mental health assessment, mobile device, mobile phone, internet technology, psychiatric disorders, mobile phone

## Abstract

**Background:**

The assessment of behaviors related to mental health typically relies on self-report data. Networked sensors embedded in smartphones can measure some behaviors objectively and continuously, with no ongoing effort.

**Objective:**

This study aims to evaluate whether changes in phone sensor–derived behavioral features were associated with subsequent changes in mental health symptoms.

**Methods:**

This longitudinal cohort study examined continuously collected phone sensor data and symptom severity data, collected every 3 weeks, over 16 weeks. The participants were recruited through national research registries. Primary outcomes included depression (8-item Patient Health Questionnaire), generalized anxiety (Generalized Anxiety Disorder 7-item scale), and social anxiety (Social Phobia Inventory) severity. Participants were adults who owned Android smartphones. Participants clustered into 4 groups: multiple comorbidities, depression and generalized anxiety, depression and social anxiety, and minimal symptoms.

**Results:**

A total of 282 participants were aged 19-69 years (mean 38.9, SD 11.9 years), and the majority were female (223/282, 79.1%) and White participants (226/282, 80.1%). Among the multiple comorbidities group, depression changes were preceded by changes in GPS features (*Time*: *r*=−0.23, *P*=.02; *Locations*: *r=*−0.36, *P<*.001), exercise duration (*r=*0.39; *P=*.03) and use of active apps (*r=*−0.31; *P*<.001). Among the depression and anxiety groups, changes in depression were preceded by changes in GPS features for *Locations* (*r*=−0.20; *P*=.03) and *Transitions* (*r*=−0.21; *P*=.03). Depression changes were not related to subsequent sensor-derived features. The minimal symptoms group showed no significant relationships. There were no associations between sensor-based features and anxiety and minimal associations between sensor-based features and social anxiety.

**Conclusions:**

Changes in sensor-derived behavioral features are associated with subsequent depression changes, but not vice versa, suggesting a directional relationship in which changes in sensed behaviors are associated with subsequent changes in symptoms.

## Introduction

### Background

Behaviors such as levels of activity and social engagement are associated with common mental health conditions such as depression and anxiety [1-4]. Retrospective evaluations of these behaviors through self-report measures or interviews rely heavily on retrospective recall, which is subject to systematic biases [5,6]. Even more accurate methods, such as ecological momentary assessment (EMA), which acquire self-reported experiences in the course of peoples’ lives [7,8], have not proven practical over extended periods outside of research settings [7]. Accelerometry data from wearable devices that measure activity levels have also been associated with depression [9], but many people stop wearing the devices within the first weeks [10].

Smartphones are becoming ubiquitous. As of 2019, 81% of Americans owned a smartphone [11], as did 76% of people in countries with advanced economies, and 45% in emerging nations [12]. Smartphones are fully integrated into our lives, supporting a growing number of activities. Smartphones contain embedded networked sensors that provide continuous, objective data without user effort, which can be used to produce behavioral markers. A growing body of research suggests that these sensor data can be associated with common mental health problems [13]. Location features derived from GPS actual measurement of patterns of locations visited, time in locations, and travel in phone communications such as text messaging and phone and app use have been associated with depression, anxiety, and social anxiety [14-17].

The potential for personal mobile sensing to improve our understanding of the relationship between behavior and mental health, as well as to advance clinical care, has been widely recognized [18]. However, although there is promise, research to date has had a number of weaknesses. Many of these studies have been conducted in small, relatively homogenous groups, such as students [15,16,19-21]. Although there has been some specificity, with location features tending to identify depression [14,15,19,22,23] and communication features tending to predict social anxiety [17], there have also been a few studies that have found the opposite [16,24]. This may be because of the heterogeneity in symptoms and comorbidities [25], which are common and have not been considered in existing research [13].

To date, studies have focused on using sensed behavioral features to estimate a person’s state, either the presence or absence of a condition or symptom severity. With some exceptions, they have generally not evaluated the capacity for sensed behavioral features to predict whether symptoms will increase or decrease in the future. Among the few studies that have examined the capacity of sensed features to predict symptom change, one small study of 18 patients with bipolar disorder found that greater inconsistencies in rates of typing on a smartphone keyboard were related to future greater depressive symptom severity [26]. Relative to studies that use sensed behavior to estimate a person’s state, the temporal relationship between sensed behaviors and symptom change has received relatively little attention.

### Objectives

In this study, we examine the temporal relationship between changes in sensor features and subsequent changes in mental health symptoms in a large sample of participants. The aim of this exploratory study is to evaluate whether changes in classes of smartphone sensor features were associated with changes in symptom severity for depression, anxiety, and social anxiety, across all participants as well as within groups clustered based on symptoms.

## Methods

### Participants

Participants were recruited from July 15 to July 26, 2019*,* through ResearchMatch, a National Institute of Health-funded volunteer network, and the Center for Behavioral Intervention Technologies research registry. Participants were included if they were US citizens and residents, age ≥18 years, could read English, and had an Android smartphone. Participants were excluded if they endorsed, via self-report, having been diagnosed with a severe mental illness, defined as bipolar disorder, schizophrenia, or other psychotic disorder. Participants were compensated for completing measures at set assessment points as well as for completing EMA check-ins. Compensation for completing assessments increased as the study period progressed, such that participants were compensated relatively less for early assessment points and relatively more for assessments toward the end of the study. No single assessment was compensated at more than US $32.50 per assessment time point. Recruitment was advertised as a study on depression and deliberately oversampled depressed participants such that at least 50% of the sample experienced at least moderate depression symptom severity according to the 8-item Patient Health Questionnaire (PHQ-8).

### Procedures

Participants downloaded the Passive Data Kit [27] mobile app, which unobtrusively collects phone sensor data and administers surveys. Web-based questionnaires were administered every 3 weeks. Participants were enrolled in the study for 16 weeks. All procedures were approved by the Northwestern University Institutional Review Board, and informed consent was obtained from all participants before participation.

### Measures

Participants completed web-based symptom severity assessments at baseline and every 3 weeks until the end of the study period (ie, weeks 4, 7, 10, 13, and 16). Symptom measures included depression severity (PHQ-8) [28], generalized anxiety disorder (Generalized Anxiety Disorder 7-item scale [GAD-7]) [29], and social anxiety disorder (Social Phobia Inventory [SPIN]) [30]. The PHQ-8 was administered as an EMA survey after the baseline assessment point and, subsequently, had one additional assessment point (week 1) relative to other symptom measures.

Phone sensor data included GPS coordinates sampled once every 5 minutes, communication information (ie, phone logs and duration, text message logs, and length), and open apps. Assessment weeks occurred every 3 weeks, during which participants were asked each evening to label the semantic location (type of location) that they had visited for more than 10 minutes [31]. A series of maps identifying each location were presented, and participants selected the category of each place (eg, home, work, errand, entertainment, place of worship, etc).

### Data Analyses

#### Phone Sensor Feature Transformation

##### Overview

We considered four categories of phone features for our analysis: GPS-derived movement and location information, semantic locations, app use, and phone-based communication (calls and texts).

To increase interpretability and reduce the number of sensor features, we aggregated features first based on their phone sensor source, as different sensor sets provide unique information. Within each sensor set, we used unit weightings that maximized the interpretability for each feature aggregation. Where possible, we used existing theory to guide our unit aggregations. All sensor features were standardized (mean-centered with unit variance) across the full sample and averaged to produce sensor groupings within the four sensor categories. A full list of features, feature calculations, and their groupings can be found in Multimedia Appendix 1.

##### GPS-Derived Location and Movement

Following the methodology of Saeb et al [20], we computed high-level features from GPS data that measure participant movement, including location variance (variability in GPS location), total unique location clusters, location entropy (variability in time spent at location clusters), normalized entropy, total distance traveled, average velocity, and circadian movement (extent to which sequence of locations followed a 24-hour pattern). The features were aggregated into *Locations* (location cluster and location variance; represents the number and variability in locations visited), *Time* (total entropy, normalized entropy, and circadian movement; represents the variability in time spent across locations), and *Transitions* (distance traveled and velocity; represents travel between locations).

##### Semantic Location

Labels for semantic location categories included home duration, work duration, shopping duration, social activities duration (eg, friends’ homes and entertainment), religious activities duration (eg, place of worship), and exercise location duration (eg, gyms). During the nonassessment weeks, semantic labels were assigned to locations visited using GPS coordinates assigned during the assessment weeks. This allowed us to estimate the daily duration of time participants spent in each semantic location category.

##### Communication

The number of incoming and outgoing calls and texts, call duration, and text message length were summed to obtain daily aggregates. The feature groups were *Calls* and *Text Messages*.

##### App Use

Apps running in the foreground of the phone were sampled every 5 minutes. We aggregated to produce daily app use duration measurements. We grouped apps of interest into 3 categories based on previous theory that certain apps facilitate active use, whereas others elicit more passive use [32,33]. This theoretical underpinning resulted in 3 categories of app use that were manually constructed using unit weighting. Final categorizations included: *Active Apps* (eg, messaging, email, and maps), which required active engagement to complete the primary essential task of each app, *Information Consumption Apps* (eg, YouTube and web browsers) where the primary purpose was more passive consumption of information or entertainment and *Social Apps* (eg, Facebook, Instagram, and Snapchat), which were considered social media apps, and were generally considered as a unique category of apps [34].

#### Population Clustering

Heterogeneity in underlying symptom patterns may impede the ability to observe clinically meaningful relationships between sensor features and symptom severity [13,35]. We used a data-driven approach, performing k-means clustering on the baseline PHQ-8, GAD-7, and SPIN items [36]. We chose k=4 using the elbow heuristic to choose the number of clusters (Multimedia Appendix 2). Qualitative analysis of these clusters showed that the 4 groups roughly corresponded to (1) a *Minimal Symptom* cluster (n=88), comprising participants characterized by low mean scores on all outcome measures; (2) a *Depression and* *Social Anxiety* cluster (n=71) that included participants with predominantly moderate severity scores on the PHQ-8 and the SPIN, but low scores on the GAD-7 measures; (3) a *Depression and Anxiety* cluster (n=69), characterized by generally moderate-severe symptoms on the PHQ-8, moderate symptoms on the GAD-7, but mild ratings on the SPIN; and a (4) *Multiple Comorbidities* cluster (n=54) characterized by elevated ratings across all three symptom measures, with a substantial proportion scoring in the severe range.

#### Statistical Methods: Correlation of Changes in Sensors to Changes in Symptom Severity

**Figure 1:**
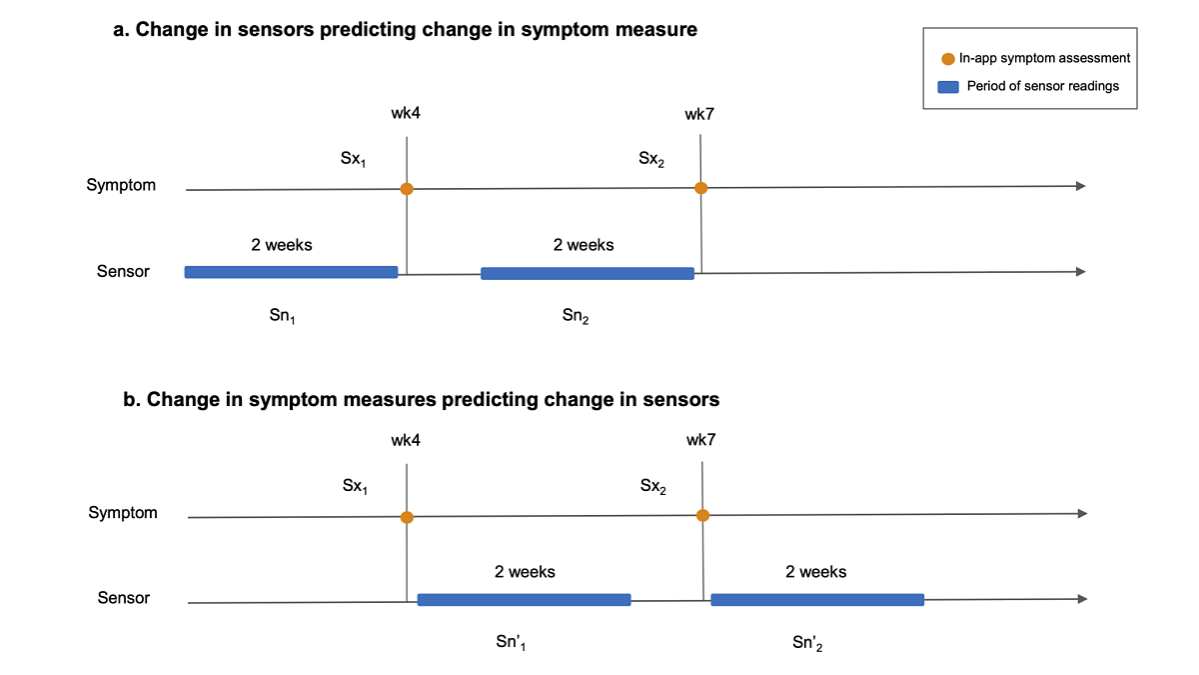
The sensor window preceding (a) and proceeding (b) the assessment check-ins. Correlations are run as corrected (Sx_2_−Sx_1_, Sn_2_−Sn_1_) and corrected (Sx_2_−Sx_1_, Sn'_2_−Sn'_1_).

Figure 1 shows the strategy we used to lag, by 2 weeks, repeated measure correlations [37] of the changes in phone sensor features with changes in symptom severity. The 2-week window for sensor features, consistent with previous research [14,20], was used to allow for sufficient quality of sensor readings to match the retrospective time spans of self-report questionnaires and to maximize data available for analysis while preventing overlapping data sources (ie, symptom outcomes and concurrent sensed behavioral data) from being used at different time points. For the PHQ-8, we had six check-ins across the entire study, yielding five pairs of changes for each participant, whereas for GAD-7 and SPIN, we had five check-ins, yielding four pairs of changes for each participant. For analyses, in which changes in sensor features were used to estimate subsequent symptom severity, Sn_2_−Sn_1_ was correlated with Sx_2_−Sx_1_. For analyses in which changes in symptom severity were used to estimate changes in subsequent sensor features, Sx_2_−Sx_1_ was correlated with Sn'_2_−Sn'_1_. To correct for multiple comparisons, we computed adjusted *P* values using the Benjamini-Hochberg procedure to control the false discovery rate [38].

If one assessment check-in was missing from a given pair of check-in dates, we used a within-person mean-fill method for the missing assessment. Any pair of assessment check-ins that had missing phone sensor data was discarded from analyses. Power calculations revealed that a sample size of 255 would be required to detect an effect size (correlation, |ρ|) of 0.2 at an α of .05 and power (β) of .90.

## Results

### Participants

**Figure 2:**
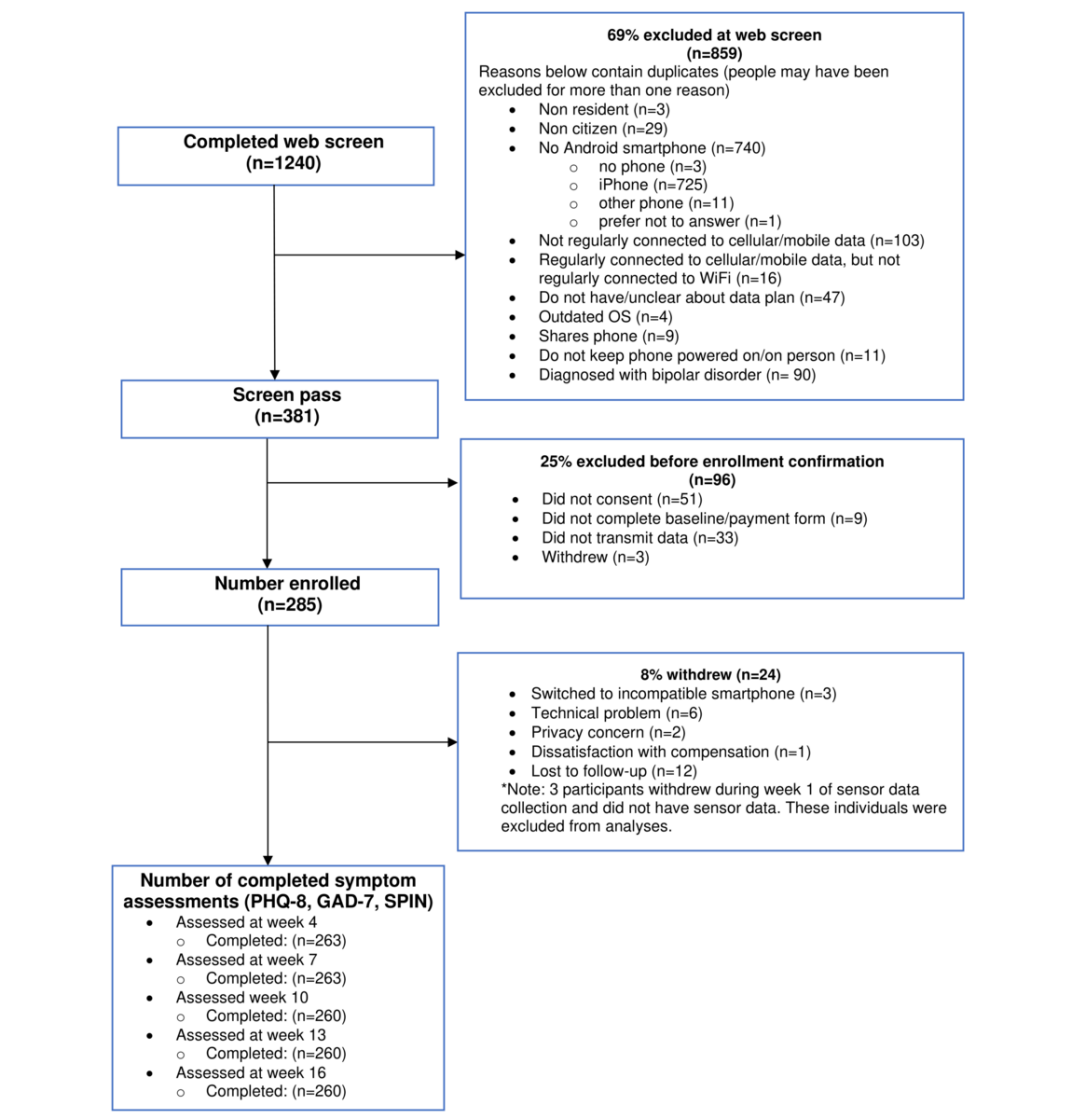
Participant flow diagram. GAD-7: Generalized Anxiety Disorder 7-item scale; PHQ-8: 8-item Patient Health Questionnaire; SPIN: Social Phobia Inventory.

The flow of participants in this study is shown in Figure 2. Participant demographic and baseline symptom severity characteristics across the entire sample and participant clusters are detailed in Multimedia Appendix 3.

### Symptom Change Over Time

Ordinary least square regression revealed no significant change in symptom severity as a function of time (PHQ-8: *P*=.80; GAD-7: *P*=.83; SPIN: *P*=.57). However, there was substantial within-participant variability depending on the symptom measure, with mean SDs of 2.66, 3.50, and 5.90, for the PHQ-8, GAD-7, and SPIN, respectively.

### Association Between Sensor-Derived Behavioral Feature Changes and Subsequent Symptom Severity Change

#### Overview

Table 1 displays the repeated measure correlations primary outcomes by symptom cluster.

**Table 1:** Repeated measure correlations between sensor and symptom changes and symptom and sensor changes^a^.

Characteristics						Repeated measure correlations																			
						Change in sensor features association with change in symptom measure											Change in symptom measure association with change in sensor features								
						Value, n		dof^b^		r_rm_^c^		*P* value (uncorrected)		*P* value (corrected)^d^		Value, n			dof^b^		r_rm_		*P* value (uncorrected)		*P* value (corrected)^d^
**Symptom measure: PHQ-8^e^**																									
	**Full sample^f^**																								
		**GPS features**																							
			Locations	223			802		−0.17		<.001		<.001		225			801		<0.001		.98		.98	
			Time	223			802		−0.12		<.001		.003		225			801		−0.006		.86		.97	
			Transitions	223			802		−0.12		<.001		.003		225			801		0.020		.56		.94	
		**Semantic location**																							
			Home duration	−225			806		0.054		.13		.23		224			801		0.017		.64		.95	
			Work duration	192			700		0.026		.50		.53		190			691		−0.012		.74		.97	
			Shopping duration	212			767		0.009		.80		.80		210			210		−0.005		.88		.97	
			Social activities duration	219			790		−0.062		.08		.17		207			207		−0.021		.57		.94	
			Religious activities duration	54			195		−0.084		.24		.36		44			44		−0.14		.08		.31	
			Exercise location duration	85			314		0.18		.001		.005		81			81		−0.13		.02		.30	
		**Communication**																							
			SMS text messages	223			796		−0.034		.34		.46		221			790		−0.062		.08		.31	
			Calls	225			802		0.03		.40		.50		221			786		−0.023		.52		.94	
		**App use**																							
			Active apps	226			809		−0.041		.24		.36		225			807		−0.004		.90		.97	
			Information consumption apps	225			805		0.026		.47		.53		226			809		−0.072		.04		.30	
			Social apps	208			748		0.073		.05		.14		207			746		−0.021		.57		.94	
	**Subgroups (features with corrected *P*≥.1 omitted)**																								
		**Multiple comorbidities**																							
			Locations	41			143		−0.36		<.001		<.001		41			143		0.021		.80		.93	
			Time	41			143		−0.23		.005		.02		41			143		−0.061		.46		.70	
			Transitions	41			143		−0.18		.03		.07		41			143		0.051		.55		.74	
			Exercise location duration	11			41		0.39		.01		.03		10			33		−0.13		.45		.70	
			Active apps	42			146		−0.31		<.001		<.001		42			146		0.10		.22		.70	
		**Depression and anxiety**																							
			Locations	56			204		−0.20		.005		.03		56			202		0.022		.75		.94	
			Transitions	56			204		−0.21		.002		.03		56			202		−0.17		.02		.22	
		**Depression and social anxiety**																							
			Locations	61			218		−0.17		.01		.08		62			218		0.007		.92		.93	
			Time	61			218		−0.16		.02		.08		62			218		0.025		.71		.93	
			Social Activities Duration	60			214		−0.17		.01		.08		57			208		0.006		.93		.93	
**Symptom measure: SPIN^g^**																									
	**Depression and social anxiety (features with corrected *P***≥.1 omitted)****																								
		Calls			66		195		0.25		<.001		.005		66			193		-0.045		.53		.73	

^a^There were no significant associations between sensor features and subsequent 8-item Patient Health Questionnaire (PHQ-8) symptoms or PHQ-8 symptoms and subsequent sensor features within the *minimal symptoms* group. There were also no significant associations between sensor features and subsequent Generalized Anxiety Disorder 7-item scale (GAD-7) symptoms or GAD-7 symptoms and subsequent sensor features for any subgroup.

^b^dof = n (k − 1), where n is the total number of participants and k is the average number of observations per participant.

^c^Repeated measures correlation coefficient.

^d^Benjamini-Hochberg corrected *P* values. Identical *P* values are due to the recursive definition of the Benjamini-Hochberg correction; it is possible for corrected *P* values to be identical, especially for nonsignificant correlations.

^e^PHQ-8: Patient Health Questionnaire-8 item.

^f^Full results are provided for Patient Health Questionnaire-8 item results in the full sample only to display all sensed behavioral features. Thereafter only relationships with corrected *P*<.1 are displayed.

^g^SPIN: Social Phobia Inventory.

#### Location Features

Patterns in individuals’ movements were associated with subsequent changes in psychological symptoms, specifically depressive symptoms. Changes in GPS-derived *Location* were negatively associated with changes in the PHQ-8 in two of three symptom clusters, *Multiple Comorbidities* (*r=*−0.36; *P*<.001), *Depression and Anxiety* (*r*=-.20; *P*=.03), and the full sample (*r=*−0.17; *P*<.001) and trended toward significance in the *Depression and Social Anxiety* cluster (*r=*−0.16; *P*=.08), but the results were not statistically significant. Changes in GPS-derived *Time* were negatively associated with changes in PHQ-8 within the *Multiple Comorbidities* cluster (*r*=−0.23; *P*=.02) and the full sample (*r*=−0.12; *P*=.003) and trended toward significance in the *Depression and Social Anxiety* cluster (*r=*−0.16; *P*=.08), but the results were not statistically significant. Changes in GPS-derived *Transitions* were negatively correlated with changes in PHQ-8 for the *Depression and Anxiety* cluster (*r=*−0.21; *P*=.03) and the full sample (*r=*−0.12; *P*=.003) and trended toward significance in the *Multiple Comorbidities* cluster (*r*=−0.18; *P*=.07), but the results were not statistically significant. There were no significant relationships for the *Minimal Symptom* cluster for the PHQ-8 and no significant relationships between GPS features and subsequent changes in the GAD-7 or SPIN.

Certain types of semantic locations were also associated with PHQ-8 changes. In the depression and social anxiety cluster, there was a trend toward significance for *Social Activity Duration*, which was negatively correlated with changes in the PHQ-8 (*r*=−0.17; *P*=.08), but the results were not statistically significant. Within the *Multiple Comorbidities* symptom cluster and the full sample, *Exercise Location Duration* was positively associated with subsequent changes in PHQ-8 scores (*Multiple Comorbidities* cluster: *r=*0.39, *P*=.03; full sample: *r=*0.18, *P*=.005). This unexpected association between changes in exercise locations and changes in PHQ-8 was likely due to a preponderance of individuals who spent no time in exercise-based locations (ie, zero change in *Exercise Location Duration* from zero time spent in exercise locations), thus overweighting the data of some individuals who saw increased time spent in exercise-based locations with increased PHQ-8. There were no significant relationships within the minimal symptom cluster for the PHQ-8, and no significant relationships were found between semantic location features and subsequent changes in the GAD-7 or SPIN.

#### Telephone Calls

Within the *Depression and Social Anxiety* baseline cluster, increases in calls were associated with increases in SPIN scores (*r*=0.25; *P<*.001).

#### App Use

Changes in *Active App Use* were negatively correlated (*r=*−0.31; *P*<.001) with changes in PHQ-8 scores within the *Multiple Comorbidities* cluster.

### Association Between Changes in Symptom Severity and Subsequent Change in Sensor-Derived Behavioral Features

There were no significant correlations between changes in any symptom severity measures and subsequent changes in sensor-derived behavioral features.

### Missing Data

Across all treatment weeks, missingness (ie, the number of missing observations/total number of possible observations for all 282 participants) was higher for the PHQ-8 (277/1692, 16.37%) assessments than for the GAD-7 (104/1410, 7.38%) and SPIN (104/1410, 7.38%). PHQ-8 assessments were administered through our smartphone app, whereas the GAD-7 and SPIN were administered through REDCap (Research Electronic Data Capture) [39]. In addition, across symptom outcome measures and relative to baseline symptom levels (Multimedia Appendix 3), those with missing assessments tended to have slightly higher baseline symptom severity (PHQ-8 range: 10.94-12.82; GAD-7 range: 9.95-11.5; and SPIN range: 24.52-27.48).

## Discussion

### Principal Findings

Changes in numerous phone sensor–derived behavioral features were associated with subsequent changes in mental health symptoms among people with elevated symptoms of depression. However, changes in symptoms were not associated with subsequent changes in behavioral features. GPS location features were fairly consistently and negatively, albeit modestly, related to subsequent changes in depression severity across symptom groups. This is consistent with a number of previous relatively small studies showing correlations between GPS features and depression [14,19,20,22,40,41]. This larger study confirms these earlier findings, and importantly, indicates a directional relationship in which GPS features are associated with subsequent increases or decreases in depressive symptoms, but not with anxiety or social anxiety.

The types of locations (work, shopping, etc) people visited were less consistently related to changes in depression. This does not necessarily mean that specific locations are unimportant at the individual level: one person may like shopping, whereas another may detest it. However, this suggests that patterns of movement through geographic space, irrespective of the destinations or locations to which one travels, are indicators of symptom change among people with depression. We speculate that this may reflect a loss of motivation expressed through geographic movement. Perhaps more speculatively, it may also be that changes in neurocognitive mechanisms, such as executive control, affect, and motivation, impact both depression and basic mechanisms involved in movement through geographic space [42,43].

The different constellations of symptoms that participants experience impact the salience of some sensed behaviors in predicting outcomes. For example, only within participants in the *Depression and Social Anxiety* symptom cluster was the amount of time spent in locations related to social activities associated with (at the trend level) subsequent change in depressive symptoms, suggesting that although locations are generally useful for depression prediction, social activities may be particularly useful when social anxiety symptoms are present. *Active App Use* (texting, email, and mapping) was associated with depression change among those with multiple and more severe comorbidities. Although GPS features were generally useful, features integrating time and location were not useful among those with comorbid generalized anxiety, and features measuring transitioning between locations were not useful for those with comorbid social anxiety. Thus, there was support for the notion that symptom constellations are important considerations for some features.

Increases in telephone calls were associated with increases in social anxiety symptoms among clusters characterized by depression and social anxiety. This finding notwithstanding, the capacity for sensor-derived features to be associated with changes in social anxiety symptoms was not as consistent as that for depressive symptoms. Furthermore, we did not find any associations between sensor-derived features and generalized anxiety symptom changes.

These findings indicate that sensor-derived behavioral features, which are objective and can be acquired with reduced participant effort, can be a useful tool for investigating the role of some behaviors in changing depressive symptom severity. There has been much speculation about the clinical potential of mobile sensing [18,44]. The effect sizes are modest, albeit consistent with many other studies that have examined the use of sensed behavioral features to estimate the presence or severity of symptoms [16,19]. This study does not support the use of phone sensor data alone in monitoring symptom changes in mental health populations; however, phone sensor data may be useful in conjunction with other networked sensing tools such as wearables. Phone sensor data may be useful for digital mental health interventions [45]. Just-in-time adaptive interventions [46,47] use individualized data to predict risk and deliver context-aware intervention material that is adaptive. These approaches are increasingly applied in digital health interventions, such as identifying when to send messages to increase physical activity such as step counts [48]. The promise of delivering motivational messages at opportune moments that reinforce behavioral activation strategies, such as visiting someplace new, spending more time outside of the home or work, or engaging socially, has the potential to improve engagement with these tools and reduce depression.

### Limitations

This study had several limitations. First, the exploratory nature of this study requires interpreting results with caution and necessitates that future work must explicitly test the a priori hypotheses arising from these results. Next, although we establish significant temporal relationships between sensed behavior changes and subsequent changes in symptom severity, our study is not experimental and does not establish causal relationships. Furthermore, our sensor feature aggregations were limited to single sensor sources and were constructed to maximize interpretability; however, future studies that use data-driven aggregations are necessary to help inform feature aggregations across sensor modalities. Although aggregating across sensor modalities presents a challenge for interpretability, future work that examines cross-sensor aggregations could yield improved estimation of sensed behaviors and, subsequently, more robust associations with changes in symptoms. In addition, although these findings provide some support for the hypothesis that sensed behavior change is associated with subsequent changes in depression and not vice versa, this study examined the associations between changes in sensor features and subsequent changes in symptom severity measures lagged by 2 weeks, and therefore should not be generalized to periods beyond the 2-week window. Another limitation is that our sample only included those who used Android devices and agreed to participate in this research. App use and communication data are not readily available for iOS devices. Regarding data missingness, across all the surveys, individuals who had missing data had higher baseline symptom severity than the overall sample, though not dramatically so; thus, data were missing not at random. These missingness rates are in line with established criteria that are often used as the standard for good trial data [49]. Finally, although we controlled for multiple analyses, we nonetheless caution against overinterpretation of more isolated findings that need to be replicated in future studies.

### Conclusions

The ubiquity of smartphones with networked sensors has opened up new opportunities to identify behavioral markers related to mental health that can be acquired continuously and effortlessly. Changes in movement through geographic space were consistently associated with subsequent changes in depressive symptoms; however, there was no evidence that changes in depression were associated with subsequent changes in sensed behaviors. This supports a directional relationship in which changes in movement patterns precede symptom change, but symptom change does not precede changes in movement behaviors.

## Appendix

Multimedia Appendix 1 Sensor features and groupings.

Multimedia Appendix 2 Symptom cluster elbow plot.

Multimedia Appendix 3 Demographics and baseline characteristics.

## Abbreviations

EMA: ecological momentary assessment
GAD-7: Generalized Anxiety Disorder 7-item scale
PHQ-8: 8-item Patient Health Questionnaire
REDCap: Research Electronic Data Capture
SPIN: Social Phobia Inventory
