# Supplementary material for: Evaluation of Changes in Depression, Anxiety, and Social Anxiety Using Smartphone Sensor Features: Longitudinal Cohort Study
Source: J Med Internet Res. 2021 Sep 3;23(9):e22844. doi: 10.2196/22844 (PMC8449302; doi:10.2196/22844)
Supplement: Multimedia Appendix 1 [file jmir_v23i9e22844_app1.pdf]

Multimedia Appendix 1. Sensor Features and Groupings

**Grouped Feature**[a]	**Derived Features**	**Raw Feature**	**Sampling Rate**	**Notes**
"Active" apps	{messaging, email, maps} app daily usage (min)	foreground application	5 min	
"Information Consumption" apps	{YouTube, web browser} daily usage (min)			
"Social" apps	{Facebook, Instagram, Snapchat} app daily usage (min)			
Home Duration	home label location duration (min)	GPS location, EMA location labels	5 min, daily	
Work Duration	{work, education} label location duration (min)			
Religious Activities Duration	religion label location duration (min)			
Exercise Location Duration	exercise label location duration (min)			
Shopping Duration	{shopping, errands} label location duration (min)			
Social Activities Duration	{another's home, entertainment, food} label location duration (min)			
SMS Communications	daily {in/out/total} SMS count	SMS message logs	event-based	
	daily {in/out/total} SMS length			
Telephone Calls	daily total call count	call logs	event-based	
	daily total call duration			
Transitions	{weekday/weekend/total} daily distance traveled	GPS locations	5 min	
	{weekday/weekend/total} daily velocity			
Locations	total location variance[b]			calculated as $log(Var[long] + Var[lat])$
	total location clusters[c]			The number of unique location clusters detected by an adaptive k-means algorithm run on stationary lat/long coordinates
Time	{normalized/raw} location entropy[d]			
	total circadian movement[e]			

[a]All features are standardized and then averaged for the final grouped feature value.

[b]*Total location variance:* calculated as $log(Var[long] + Var[lat])$ over the designated sensor window.

[c]*Total location clusters:* the number of unique location clusters detected by an adaptive k-means algorithm[21] run on stationary lat/long coordinates over the designated sensor window.

[d]*Location entropy:* entropy calculated as $Entropy = -\sum_{i=1}^{N} p_i \log(p_i)$
where p_i is the percentage of time spent at detected location *i* over the *N* total detected locations. Normalized entropy is entropy divided by $log(N)$. This matches the "entropy" calculation presented in Saeb et al. 2016.

[e]*Total circadian movement:* calculated as the amount of location "energy" that fell into bins of 24 hrs ± 0.5 hrs via power spectral density[21]

$$Energy = \frac{1}{i_U - i_L} \sum_{i=i_L}^{i_U} psd(f_i)$$

where $psd(f_i)$ is the power spectral density at frequency f_i , i_U the upper bound of the frequency range corresponding to 24.5 hours, and i_L the lower bound of the frequency range corresponding to 23.5 hours. This energy measure captures the "periodicity" of the signal in terms of roughly 24 hour cycles. *Energy* is calculated separately for location
