# Supplementary material for: Evaluation of Changes in Depression, Anxiety, and Social Anxiety Using Smartphone Sensor Features: Longitudinal Cohort Study
Source: J Med Internet Res. 2021 Sep 3;23(9):e22844. doi: 10.2196/22844 (PMC8449302; doi:10.2196/22844)
Supplement: Multimedia Appendix 2 [file jmir_v23i9e22844_app2.pdf]

Multimedia Appendix 2. Symptom Cluster Elbow Plot

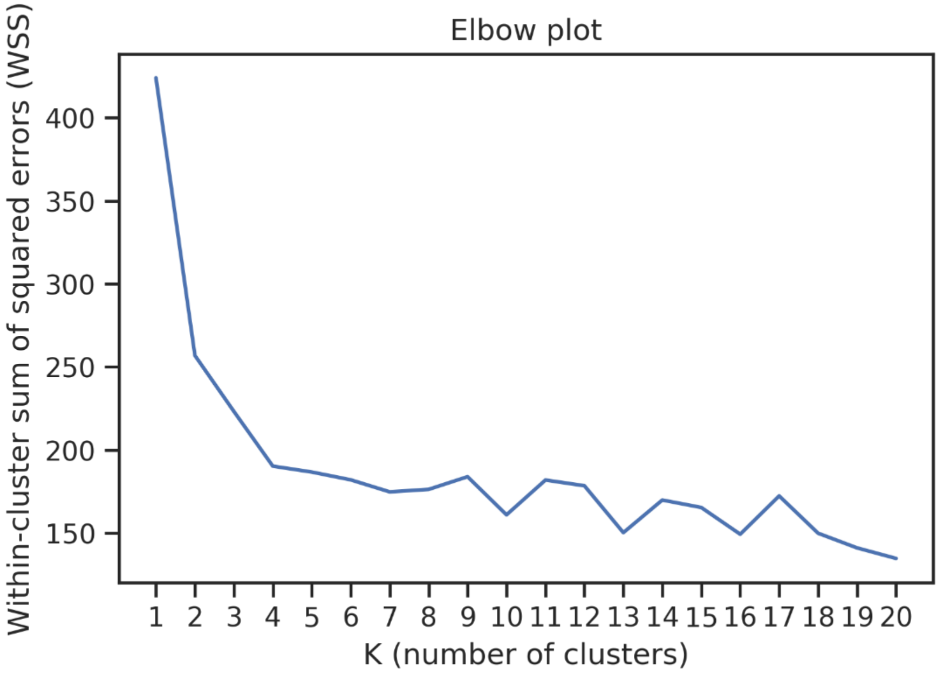

We use the “elbow heuristic” to determine the number of clusters we used. We plot the within-cluster sum of squared errors (WSS), which measures how far all of the points assigned to a cluster are from the cluster center. As we increase the number of clusters, the WSS is likely to further decrease, so we want to balance between minimizing the WSS and subdividing our participant population too finely. To achieve this balance, we follow the common practice of selecting K to be the value at which the WSS is minimized with the lowest value of K (the “elbow”).
