# Supplementary material for: Evaluation of Changes in Depression, Anxiety, and Social Anxiety Using Smartphone Sensor Features: Longitudinal Cohort Study
Source: J Med Internet Res. 2021 Sep 3;23(9):e22844. doi: 10.2196/22844 (PMC8449302; doi:10.2196/22844)
Supplement: Multimedia Appendix 3 [file jmir_v23i9e22844_app3.pdf]

Multimedia Appendix 3. Demographics and Baseline Characteristics

Variable	Total (*n*=282)	Minimal Symptoms (*n*=88)	Depression and Generalized Anxiety (*n*=69)	Depression and Social Anxiety (*n*=71)	Multiple Comorbidities (*n*=54)
Age, mean (sd)	38.9 (11.9)	40.7 (13.4)	39.8 (11.4)	38.3 (11.5)	35.9 (10.3)
Sex (assigned at birth), n (%)					
Female	223 (79.1%)	68 (77.3%)	54 (78.3%)	53 (74.6%)	48 (88.9%)
Male	59 (20.9%)	20 (22.7%)	15 (21.7%)	18 (25.3%)	6 (11.1%)
Gender identity, n (%)					
Woman	212 (75.2%)	68 (77.3%)	50 (72.5%)	48 (67.6%)	46 (85.2%)
Man	59 (20.9%)	20 (22.7%)	15 (21.7%)	18 (25.4%)	6 (11.1%)
Non-binary	7 (2.5%)	0 (0%)	1 (1.4%)	4 (5.6%)	2 (3.7%)
Other	4 (1.4%)	0 (0%)	3 (4.3%)	1 (1.4%)	0 (0%)
Race, n (%)					
Black/African American	21 (7.4%)	12 (13.6%)	4 (5.8%)	3 (4.2%)	2 (3.7%)
Native American/Alaskan Native	3 (1.1%)	1 (1.1%)	2 (2.9%)	0 (0%)	0 (0%)
Asian	9 (3.2%)	2 (2.3%)	1 (1.4%)	2 (2.8%)	4 (7.4%)
White	226 (80.1%)	63 (71.6%)	57 (82.6%)	60 (84.5%)	46 (85.2%)
More than one Race	21 (7.4%)	8 (9.1%)	5 (7.2%)	6 (8.5%)	2 (3.7%)
Declined to answer	2 (0.7%)	2 (2.3%)	0 (0%)	0 (0%)	0 (0%)
Ethnicity, n (%)					
Hispanic/Latinx	27 (9.6%)	14 (15.9%)	5 (7.2%)	4 (5.6%)	4 (7.4%)
Non-Hispanic/Non-Latinx	253 (89.7%)	73 (83%)	64 (92.8%)	66 (93%)	50 (92.6%)
Unknown or declined to answer	2 (0.7%)	1 (1.1%)	0 (0%)	1 (1.4%)	0 (0%)
Education (highest level completed), n (%)					
Some high School, no diploma or GED	3 (1.1%)	0 (0%)	1 (1.4%)	1 (1.4%)	1 (1.9%)
High school/GED	13 (4.6%)	2 (2.3%)	3 (4.3%)	4 (5.6%)	4 (7.4%)
Some college, no degree	47 (16.7%)	10 (11.4%)	13 (18.8%)	12 (16.9%)	12 (22.2%)
Associates degree	39 (13.8%)	8 (9.1%)	12 (17.4)	9 (12.7%)	10 (18.5%)
Bachelor's degree	99 (35.1%)	33 (37.5%)	24 (34.8%)	25 (35.2%)	17 (31.5%)
Graduate degree	81 (28.7%)	35 (39.8%)	16 (23.2%)	20 (28.2%)	10 (18.5%)
Marital status, n (%)					
Single/never married	101 (35.8%)	34 (38.6%	22 (31.9%)	28 (39.4%)	17 (31.5%)
Domestic partnership/cohabitating	47 (16.7%)	15 (17%)	11 (15.9%)	12 (16.9%)	9 (16.7%)
Married	92 (32.6%)	27 (30.7%)	22 (31.9%)	23 (32.4%)	20 (37%)
Separated	6 (2.1%)	2 (2.3%)	1 (1.4%)	1 (1.4%)	2 (3.7%)
Divorced	33 (11.7%)	10 (11.4%)	12 (17.4%)	5 (7.4%)	6 (11.1%)
Unknown or declined to answer	3 (1.1%)	0 (0%)	1 (1.4%)	2 (2.8%)	0 (0%)
Number of people in household, n (sd)	2.5 (1.3)	2.3 (1.4)	2.6 (1.3)	2.6 (1.4)	2.6 (1.1)
Household income, n (%)					
<$20,000	33 (11.7%)	5 (5.7%)	11 (15.9%)	9 (12.7%)	8 (14.8%)
$20,000-$39,999	44 (15.6%)	18 (20.5%)	6 (8.7%)	10 (14.1%)	10 (18.5%)
$40,000-$59,999	59 (20.9%)	17 (19.3%)	16 (23.2%)	15 (21.1%)	11 (20.4%)
$60,000-$99,999	88 (31.2%)	27 (30.7%)	21 (30.4%)	23 (32.4%)	17 (31.5%)
>$100,000	49 (17.4%)	21 (23.9%)	11 (15.9%)	11 (15.5%)	6 (11.1%)
Unknown or declined to answer	9 (3.2%)	3 (3.4%)	4 (5.8%)	0 (0%)	2 (3.7%)
Employment status, n (%)					
Employed	218 (77.3%)	73 (83%)	50 (72.5%)	58 (81.7%)	37 (68.5%)
Unemployed	23 (8.2%)	3 (3.4%)	6 (8.7%)	4 (5.6%)	10 (18.5%)
Disability	17 (6%)	3 (3.4%)	7 (10.1%)	5 (7.0%)	2 (3.7%)
Retired	10 (3.5%)	7 (8%)	2 (2.9%)	1 (1.4%)	0 (0%)
Other (e.g., caregiver)	13 (4.6%)	2 (2.3%)	4 (5.8%)	3 (4.2%)	4 (7.4%)
Declined to answer	1 (0.4%)	0 (0%)	0 (0%)	0 (0%)	1 (1.9%)
Current counselling/therapy in past 4 weeks, n (%)					
Yes	102 (36.2%)	17 (19.3%)	31 (44.9%)	28 (39.4%)	26 (48.1%)
No	180 (63.8%)	71 (80.7%)	38 (55.1%)	43 (60.6%)	28 (51.9%)
Current prescribed psychotropic medication use, n (%)					
Yes	162 (57.4%)	33 (37.5%)	47 (68.1%)	48 (67.6%)	34 (63%)
No	120 (42.6%)	55 (62.5%)	22 (31.9%)	23 (32.4%)	20 (37%)
PHQ-8[a] (at baseline), mean (sd)	10.9 (6.1)	4.9 (3.8)	15.0 (3.6)	9.8 (3.9)	17.1 (3.7)
GAD-7[b] (at baseline), mean (sd)	8.8 (8.1)	2.9 (2.5)	12.5 (3.7)	6.5 (2.8)	16.7 (12.9)
SPIN[c] (at baseline), mean (sd)	28.0 (63.3)	8.5 (5.8)	21.7 (8.7)	30.1 (8.1)	65.2 (137.6)

[a]PHQ-8= Patient Health Questionnaire (8-item version)

[b]GAD-7=Generalized Anxiety Disorder 7-item questionnaire

[c]SPIN=Social Phobia Inventory
